# Loss of Fractalkine Signaling Exacerbates Axon Transport Dysfunction in a Chronic Model of Glaucoma

**DOI:** 10.3389/fnins.2016.00526

**Published:** 2016-11-24

**Authors:** Kevin T. Breen, Sarah R. Anderson, Michael R. Steele, David J. Calkins, Alejandra Bosco, Monica L. Vetter

**Affiliations:** ^1^Departments of Neurobiology and Anatomy, University of UtahSalt Lake City, UT, USA; ^2^Department of Ophthalmology and Visual Sciences, Vanderbilt UniversityNashville, TN, USA

**Keywords:** retina, glaucoma, microglia, macrophage, retinal ganglion cell, neurodegeneration, Cx3cr1, DBA/2J

## Abstract

Neurodegeneration in glaucoma results in decline and loss of retinal ganglion cells (RGCs), and is associated with activation of myeloid cells such as microglia and macrophages. The chemokine fractalkine (FKN or Cx3cl1) mediates communication from neurons to myeloid cells. Signaling through its receptor Cx3cr1 has been implicated in multiple neurodegenerative diseases, but the effects on neuronal pathology are variable. Since it is unknown how FKN-mediated crosstalk influences RGC degeneration in glaucoma, we assessed this in a chronic mouse model, DBA/2J. We analyzed a DBA/2J substrain deficient in Cx3cr1, and compared compartmentalized RGC degeneration and myeloid cell responses to those in standard DBA/2J mice. We found that loss of FKN signaling exacerbates axon transport dysfunction, an early event in neurodegeneration, with a significant increase in RGCs with somal accumulation of the axonal protein phosphorylated neurofilament, and reduced retinal expression of genes involved in axon transport, Kif1b, and Atp8a2. There was no change in the loss of Brn3-positive RGCs, and no difference in the extent of damage to the proximal optic nerve, suggesting that the loss of fractalkine signaling primarily affects axon transport. Since Cx3cr1 is specifically expressed in myeloid cells, we assessed changes in retinal microglial number and activation, changes in gene expression, and the extent of macrophage infiltration. We found that loss of fractalkine signaling led to innate immune changes within the retina, including increased infiltration of peripheral macrophages and upregulated nitric oxide synthase-2 (Nos-2) expression in myeloid cells, which contributes to the production of NO and can promote axon transport deficits. In contrast, resident retinal microglia appeared unchanged either in number, morphology, or expression of the myeloid activation marker ionized calcium binding adaptor molecule 1 (Iba1). There was also no significant increase in the proinflammatory gene interleukin 1 beta (Il1β). We conclude that loss of fractalkine signaling causes a selective worsening of axon transport dysfunction in RGCs, which is linked to enhanced Nos-2 expression in myeloid cells. Our findings suggest that distinct mechanisms may contribute to different aspects of RGC decline in glaucoma, with axonal transport selectively altered after loss of Cx3cr1 in microglia and/or macrophages.

## Introduction

The myeloid innate immune system has long been of interest in the field of neurodegeneration, not only as a responder to neuronal stress and damage but also as a driver of neurodegeneration (Ilieva et al., 2009; Czeh et al., 2011). This is also the case for diverse neurodegenerative ocular diseases, including glaucoma (Karlstetter et al., 2015). In glaucoma, retinal ganglion cells (RGCs) progressively degenerate, with the different neuronal compartments (soma, axon, dendrites, and synapses) declining at different times and potentially by different mechanisms (Libby et al., 2005a; Whitmore et al., 2005; Conforti et al., 2007; Howell et al., 2007; Calkins, 2012; Fernandes et al., 2014). In addition, multiple studies in human and experimental glaucoma suggest a link between the progressive deterioration of RGCs and alterations of glial cell types (Yuan and Neufeld, 2001; Naskar et al., 2002; Neufeld and Liu, 2003; Nakazawa et al., 2006; Inman and Horner, 2007; Bosco et al., 2011, 2015b; Lye-Barthel et al., 2013; Chong and Martin, 2015). Microglia are the resident innate immune cells of the central nervous system, and in both glaucoma patients and in animal models of glaucoma, they display increased reactivity and cluster at the optic nerve head, which is a site of early damage (Bosco et al., 2011; Yuan and Neufeld, 2001; Roh et al., 2012; Soto and Howell, 2014). It has also been demonstrated that peripheral macrophages infiltrate the retina in animal models of glaucoma (Howell et al., 2012) suggesting that different myeloid cell types may participate in glaucoma progression. However, it remains unclear how microglia and macrophages affect RGC compartmentalized degeneration in glaucoma.

In order to address the role of myeloid cells in neurodegeneration, many studies have manipulated the fractalkine (FKN) signaling system, which serves as a major neuron-to-myeloid cell communication system (Hoarau et al., 2011; Limatola and Ransohoff, 2014). The FKN receptor, Cx3cr1, is specifically expressed by cells of the myeloid lineage, including microglia, while the FKN ligand, Cx3cl1, is highly expressed by neurons (Jung et al., 2000; Cook et al., 2001). Multiple studies have suggested that Cx3cl1/Cx3cr1 signaling functions to constrain microglial activation, and thus limit potentially neurotoxic effects (Cardona et al., 2006; Limatola and Ransohoff, 2014). However, it is now appreciated that diverse myeloid functions are regulated by FKN signaling, including cytokine secretion, microglial activation, phagocytosis, process dynamics, migration, survival of subsets of circulating monocytes, and infiltration of macrophages positive for chemokine C-C motif receptor 2 (Ccr2) (Cardona et al., 2006; Lee et al., 2008; Landsman et al., 2009; Liang et al., 2009; Sennlaub et al., 2013). Given these complex roles, it is not surprising that loss of FKN signaling has diverse and sometimes contradictory effects on neurodegeneration (Wolf et al., 2013). For example, loss of FKN signaling simultaneously reduces amyloid-β burden and increases tau pathology in the same mouse model of Alzheimer's disease (Lee et al., 2014). These findings suggest that myeloid cells may differentially influence distinct aspects of neurodegeneration. However, the role of these cells is still incompletely understood, particularly for progressive age-related neurodegeneration including glaucoma.

The DBA/2J mouse models key aspects of neurodegeneration in human glaucoma because the decline of RGCs occurs in an age-related and asynchronous manner with variable onset (John et al., 1998; Anderson et al., 2002; Jakobs et al., 2005; Libby et al., 2005a). Multiple quantitative readouts have been established demonstrating progressive compartmental degeneration of RGCs in the DBA/2J retina and optic nerve. These include early downregulation of RGC genes such as the transcription factor Brn3b, accumulation of axonal proteins such as phosphorylated neurofilament (pNF) within RGC somata as transport declines, and loss of axons with subsequent gliosis in the optic nerve (Libby et al., 2005a,b; Schlamp et al., 2006; Buckingham et al., 2008; Soto et al., 2008; Dengler-Crish et al., 2014; Bosco et al., 2015b; Cooper et al., 2016). Furthermore, alterations of both resident microglia and infiltrating macrophages have been documented in this model, although the impact of these cells on RGC degeneration remains poorly understood (Mo et al., 2003; Anderson et al., 2008; Bosco et al., 2011, 2015b; Howell et al., 2012).

To address this, we sought to manipulate microglia/macrophage function and assess the effects on RGC neurodegeneration. We therefore analyzed the retinas of 10–11 month-old DBA/2J mice lacking the FKN receptor, Cx3cr1, for neuroprotective, or deleterious effects in the retina and optic nerve. Thus, we measured the density of Brn3-positive nuclei and somal pNF-positive cells across whole retinas, and analyzed the respective optic nerves for the extent of glial coverage as a proxy for axon dropout. In these same samples, we assessed changes in myeloid cells in the retina by quantifying cell densities of myeloid cell populations and assessing microglial activation by Iba1 immunostaining. We then performed flow cytometry to assess infiltration of CCR2+ macrophages and measured gene expression changes by qRT-PCR. We show that loss of fractalkine signaling in myeloid cells increases infiltration of Ccr2+ macrophages into the DBA/2J retina, selectively worsens axon transport dysfunction in RGCs, and is associated with selective changes in the expression of genes related to axon transport deficits.

## Materials and methods

### Mice

Cx3cr1^gfp/gfp^ DBA/2J mice were generated by breeding existing Cx3cr1^gfp/+^ DBA/2J mice to homozygosity (Bosco et al., 2015b). DBA/2J mice were obtained from Jackson Labs, bred in-house, and were refreshed with new breeders every 3–4 generations. Both male and female mice were used for analysis (*n* = *36* Cx3cr1^gfp/gfp^ DBA/2J mice, 21 male, 15 female; *n* = *31* DBA/2J mice, 15 male, 16 female). Mice from both strains were aged to 10–11 months, when a majority of DBA/2J eyes typically show clear glaucoma pathology (Libby et al., 2005a). Although animals of mixed gender were used, we have excluded gender as a contributing factor, since our findings were confirmed by analysis of data from male animals alone (data not shown). Experiments and mouse care were performed in compliance with the ARVO Statement for the Use of Animals in Ophthalmic and Vision Research, and with the guidelines of the University of Utah Institutional Animal Care and Use Committee.

### Tissue collection

Perfusion and dissection of retinal tissue and optic nerves for both immunohistochemistry and RNA collection were performed as previously described (Bosco et al., 2011). Briefly, retinas were dissected fresh under RNAase-free conditions for RNA isolation, or dissected from transcardially perfused mice as whole mounts, which were stored in chilled 0.1 M phosphate-buffered saline (PBS) overnight at 4°C and utilized the next day for immunohistochemistry. Also, retinal eyecups were cryoprotected, cast in gelatin blocks that were stored at −80°C, and cryosectioned radially at 16 μm thickness for immunohistochemistry. Optic nerves were postfixed, embedded in resin and prepared as 1–2 μm-thick cross-sections for toluidine blue and paraphenylenediamine (PPD) staining (Bosco et al., 2015b).

### Immunohistochemistry

Triple immunofluorescence staining was performed using established protocols and antibodies, as previously described in detail (Bosco et al., 2011). Retinal whole mount and cryosections were incubated with primary antibodies for 3 days at 4°C and incubated with secondary antibodies for 2 h. Primary antibodies used in this study included: goat anti-Brn3 (Santa Cruz sc-6026 at 1:50), mouse anti-phosphorylated neurofilament (pNF; including NFM and NFH; Dako M0762 at 1:100), rabbit anti-Iba1 (Wako at 1:1000), rat anti-CCR2 APC conjugated (R&D Systems FAB5538A at 1:10), and rabbit anti-cleaved caspase 3 (BD Biosciences BDB559565 at 1:500). pNF staining co-labeled with rabbit polyclonal neurofilament heavy antibody (Biolegend 801703) and in separate experiments was absent when primary antibody was omitted. All Alexa Fluor-conjugated (488, 568, or 647 nm) donkey secondary antibodies were used at 1:400 (Invitrogen); Alexa-Fluor 594-conjugated donkey anti rat secondary was used at 1:200 (Jackson ImmunoResearch Lab).

### *In situ* hybridization

Antisense and sense 3′UTR Dig labeled probes were generated from pCMV-SPORT6 Cx3cl1 (MGC 5859) obtained from ATCC. Probes were synthesized and hybridized using standard procedures as in Soto et al. (2008) without the use of proteinase K. Briefly, mice were perfused, eyes cast in gelatin, and 16 um radial cryosections were generated as described in their respective sections. Bound probes were detected using 1:300 Anti-Dig AP antibody (Sigma 11093274910) overnight at 4°C with 30 min color development using NBT/BCIP tablets (Sigma 11697471001).

### Microscopy

All retinal samples were imaged on an inverted confocal microscope (Nikon A1 with NIS-Elements software 4.2) using a 20x objective and resonance scanning (Bosco et al., 2012). Entire retinas were imaged using a multipoint acquisition macro of the software, collecting 25 × 25 fields at 60x magnification (0.41 μm/pixel), each spanning 30–40 μm of the retinal inner surface through a step size of 0.8 μm. Maximum intensity projections of stitched, high-resolution images were then generated for each retina, and all images were identically and minimally adjusted for brightness and contrast for analysis. Optic nerves were imaged on a compound BX51 Olympus light microscope using a 60X objective and analyzed using cellSens software as 36 high-resolution multipoint images (Bosco et al., 2015b).

### Quantification of Brn3+ nuclei

Brn3+ nuclei, corresponding to RGCs expressing Brn3a and Brn3b (Bosco et al., 2011), were sampled within the central 1.77 μm^2^ of the retina by dividing a circle centered on the optic disk into eight parts (2 dorsal, 2 ventral, 2 nasal, and 2 temporal). A 250 × 250 μm box was placed in each of these areas with blood vessels used as landmarks. RGC degeneration in the DBA/2J model is sectorial (Jakobs et al., 2005; Howell et al., 2007), so to avoid bias, when two sectors were visible within an eighth of the retina being analyzed, two boxes were placed one in each sector. The total number of Brn3+ nuclei were tallied and normalized to the area sampled (~0.06 mm^2^ per box). To be counted, nuclei had to be spherical/elliptical in shape and >1.5x the intensity of the background. Debris was uniformly more intense than Brn3+ nuclei and was excluded from the quantification. Retinas were then sorted from highest to lowest Brn3+ nuclei density. The distribution of densities of Brn3+ nuclei was not normally distributed therefore a Wilcoxon rank sum test was used to assess significance.

### Quantification of somal pNF+ RGCs

Cell somas with accumulated phosphorylated neurofilament (pNF) were quantified in the central 1.77 μm^2^ of the retina, as described for Brn3. RGC somata were identified based on shape (reflecting either somatic or somato-dendritic accumulation), size being >10 μm in diameter and fluorescence intensity being >1.5x the background. Somal pNF positive RGCs were verified to be negative for Iba1. RGCs positive for both Brn3 and somal pNF or just somal pNF were quantified. Retinas were then sorted from lowest to highest somal pNF+ RGC density. The distribution of densities of pNF+ cells was not normally distributed therefore a Wilcoxon rank sum test was used to assess significance.

### Quantification of Iba1+ cells

Three categories of Iba1+ cell were determined based on morphology: branched Iba1+ cells with a small cell soma and more than two branches, perivascular Iba1+ cells having two main branches at polar opposite sides of the cell, and amoeboid Iba1+ cells having no branches. Branched Iba1+ and perivascular Iba1+ cells were quantified in the central 1.77 um^2^ of the retina, as described for Brn3, excluding the optic disk. Amoeboid Iba1+ cells, being fewer in number, were quantified in the entire retina, excluding the optic disk and the lumen of blood vessels. Retinas were then sorted from lowest to highest density of the respective Iba1+cell morphological class. The distribution of densities of perivascular and amoeboid Iba1+ cells was not normally distributed and was analyzed by Wilcoxon rank sum test. The distribution of branched Iba1+ cells was normally distributed and was analyzed by Student's *t*-test.

### Optic nerve histopathology

Optic nerve cross-sections from the myelinated portion of the nerve, 1–1.5 mm proximal to the lamina, were embedded and 1 μm sections were cut on an ultramicrotome with a glass knife then transferred to slides. Sections were stained with PPD (Libby et al., 2005a) for 28 min using a modified protocol as previously described (Bosco et al., 2015a,b), and mounted under coverslips with Permount (Fisher).

### Quantification of non-axonal area in the proximal optic nerve

The relative area devoid of axons or dystrophic axons in individual optic nerve cross-sections was measured by segmentation of glial cells and/or glial scar and extracellular matrix from 8-bit stitched RGB images of PPD stained nerves, as previously described (Bosco et al., 2015a,b, 2016). The green channel of the RGB image provided the greatest contrast to segment glial area from non-glial elements including axons and blood vessels. Briefly, the green channel of RGB images minimally adjusted for contrast (0–10 on a scale to 0–100) and sharpness (Gauss Laplace between 1.0 and 1.1 on a scale of 0–2), were thresholded to generate a binary mask encompassing the total cross-sectional area of the optic nerve, excluding meninges and blood vessels. The axon-free nerve relative area was then calculated by determining the percent of the total optic nerve that is covered by non-axonal cells or matrix. Optic nerves were then sorted from lowest to highest percent non-axonal area. The distribution of percent optic nerve covered by non-axonal elements was not normally distributed and was analyzed by Wilcoxon rank sum test. To verify this analysis, a subset of 24 DBA/2J and 24 Cx3cr1^gfp/gfp^ DBA/2J PPD-stained optic nerves were blindly scored using a three-category system as mild, moderate, or severe, as in Libby et al. (2005a). Mild degeneration consisted of nerves with no or very little loss of axons and no gliosis. Moderate degeneration consisted of little to no gliosis but a clear presence of dystrophic axons, while a majority remained healthy. Severe degeneration consisted of nerves with clear and frequently extensive gliosis and a majority of dystrophic axons with clear and significant loss of axons overall. No significant difference was found between the genotypes with a chi-squared value of 0.93.

### Fluorescence-activated cell sorting (FACS) and flow cytometry

Four female DBA/2J or three Cx3cr1^gfp/gfp^ DBA/2J female mice at 12 months of age were perfused with saline. Retinas were dissociated in PBS, 50 mM HEPES, 0.05 mg/ml DNaseI (Sigma D4513), 0.025 mg/ml Liberase (Sigma 5401119001) for 35 min with intermediate trituration. Cells were passed through a 70 μm nylon cell strainer, washed with staining buffer (1X PBS, 2% BSA, 0.1% sodium azide, 0.05% EDTA), and red blood cells were lysed (eBioscience 00-4333-57). Cell counts were determined using a hemocytometer and Fc block (BD Biosciences 553142) was added 2 μl per 10^6^ cells. Antibodies were applied for 30 min on ice (BV421-CD45 563890, PE-CD11b 553311, APC-Ccr2 FAB5538A100). Cells were washed, pelleted, and resuspended in 500 μl staining buffer. FACS was performed using a BD FACSAria cell sorter. CD11b^+^, CD45^+^, CCR2 ± were sorted directly into RLT buffer (Qiagen 79216) and stored at −20°C. RNA from sorted cells was purified using an RNeasy Plus Micro kit (Qiagen 74034) and reverse transcribed using SuperScript IV Reverse transcriptase (Invitrogen 18090019). 832,000–937,000 events were collected for flow analysis using BD FACSDiva software. CCR2^+^ populations were analyzed gating on live CD11b^+^ CD45^+^ cells. Unstained retinas and spleens stained for CD11b, CD45 and CCR2 from the same animals were used as negative and positive controls, respectively.

### Quantitative RT-PCR

Sample preparation, cDNA synthesis, and quantitative reverse transcriptase polymerase chain reaction (qRT-PCR) were performed as previously described (Bosco et al., 2011). Briefly, whole retinas were aspirated through a 22-gage needle and RNA was isolated using a Qiagen RNeasy micro kit according to manufacturer's instructions. The mRNA quality was determined on an Agilent Bioanalyzer and samples with RNA integrity numbers <8 were discarded. First strand cDNA was synthesized using an Invitrogen Superscript III First Strand cDNA synthesis kit according to manufacturer's recommendations, and quantities determined on a Nano-drop spectrophotometer. qRT-PCR was performed on an Applied Biosystems 7900 HT instrument with QuantStudio 12 K Flex software using an Invitrogen Platinum Sybr Green qPCR supermix-UDG kit according to manufacturer's instructions. For analysis of whole retina samples (all genes except Nos-2 and interleukin 1β (Il1β)), a standard curve was generated based on five serial one-half dilutions of pooled cDNAs from a large number of 1–12-month DBA/2J retinas, and genes were analyzed in the linear range based on these curves. For Nos-2 and Il1β, we used the ΔΔCt method to calculate the relative fold change in gene expression (Livak and Schmittgen, 2001), and statistical analysis was performed using Student's *t*-test on all genes. Analyzed genes were normalized to glyceraldehyde 3-phosphate dehydrogenase (Gapdh). For statistical analysis, genes that did not amplify were set to 40 cycles, which is the limit of detection.

Primers used 5′–3′:

  GapdhF: TGCACCACCAACTGCTTAGC
  GapdhR: GGCATGGACTGTGGTCATGAG
     Iba1F: CCTGATTGGAGGTGGATGTCA
     Iba1R: GGCTCACGACTGTTTCTTTTTTCC
    Kif1bF: TTATTGATACATCCATGGGGTC
    Kif1bR: TCTCCTGAATACTGGTCACA
  Atp8a2F: CTTTGTGTTTTGTTTTCCCCGC
  Atp8a2R: CGCTGTACTTGGCCGTACTGA
    Mfge8F: GGATAATCAGGGCAAGATCA
    Mfge8R: TAGGACGCCACATACTGGAT
      Ccr2F: AAGGAGCCATACCTGTAAATGC
      Ccr2R: ATGCCGTGGATGAACTGAGG
       Ccl2F: CACTCACCTGCTGCTACTCA
      Ccl2R: GCTTGGTGACAAAAACTACAGC
   Nos-2F: TCTTGGAGCGAGTTGTGGATT
   Nos-2R: CAGCCTCTTGTCTTTGACCCA
       Il1βF: ACATCAGCACCTCACAAGCAGAG
       Il1βR: TGGGGAAGGCATTAGAAACAGTC

### Intraocular pressure (IOP)

IOP was determined as previously described (Bosco et al., 2011) with the exception of Isoflurane being used as anesthesia instead of Avertin. Briefly, mice were anesthetized using Isoflurane (2% delivered in 2 L/min oxygen) and IOP was measured using a Tonolab (Colonial Medical Supply) positioned to strike the center of the eye. Twelve measurements were collected per eye before noon, the top and bottom measurement discarded, and the remainder averaged with a SEM <0.7. By assessing mice at 3, 6, and 9 mo of age (*n* = 24, 14, and 28 eyes respectively), we confirmed that in Cx3cr1^gfp/gfp^ DBA/2J mice there was no enhancement of the existent elevation in IOP documented in DBA/2J mice (Libby et al., 2005a) and iris atrophy was present (data not shown).

### Statistics

Statistical analyses for each method are described in their respective sections. Normality of the data was examined both by histogram distribution and by percentage of data falling 1 and 2 standard deviations away from the mean. When data were not normally distributed, we created a macro in Excel to run a Wilcoxon rank sum test to determine whether the rank of the population means were significantly different. Kolmogorov-Smirnov test was performed by utilizing an online tool developed at St. John's University (retrieved from http://www.physics.csbsju.edu/stats/KS-test.html).

## Results

### Loss of Cx3cr1 exacerbates RGC axon dysfunction in the DBA/2J retina

**Figure 1:**
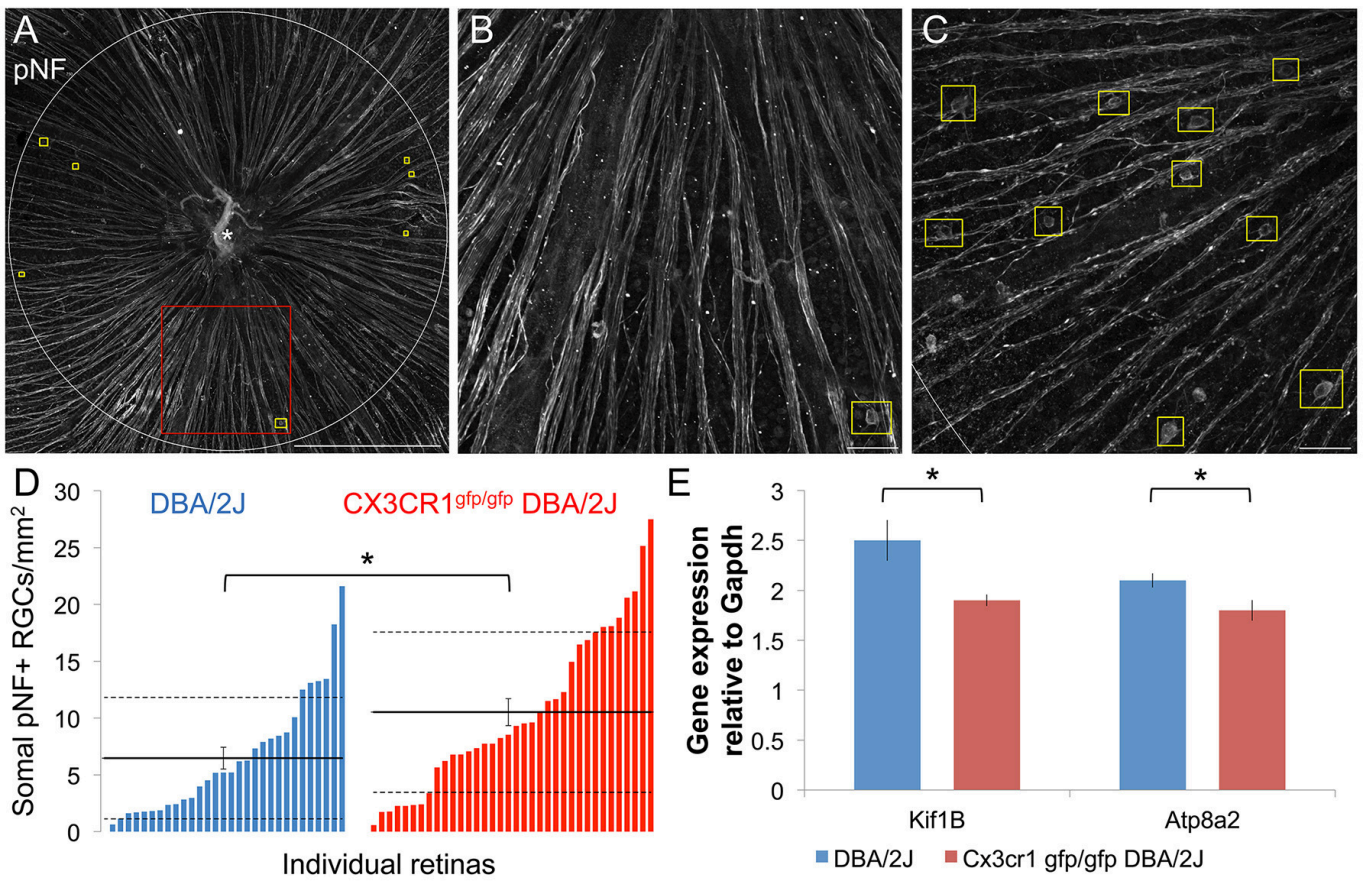
**More RGCs have somal accumulation of pNF and axonal transport genes are downregulated with loss of fractalkine signaling. (A)** Confocal image of a Cx3cr1^gfp/gfp^ DBA/2J retinal flat mount immunostained for phosphorylated-neurofilament (pNF), depicting the sampled central retinal area (1.77 mm^2^). This retina has few somal pNF+ RGCs and smooth, fasciculated axons. Yellow boxes indicate cells that show pNF accumulated within the cell soma. **(B)** Higher magnification view of an area in A (red box), depicting a cell with accumulated somal pNF. **(C)** Higher magnification of the central retinal area from a different Cx3cr1^gfp/gfp^ DBA/2J retina (same dimensions as in **B**) with abundant somal pNF+ RGCs as well as beaded and defasciculated axons. **(D)** Distribution of the number of cells with somal pNF within the central retina in DBA/2J (blue; *n* = 31 retinas) and Cx3cr1^gfp/gfp^ DBA/2J (red; *n* = 36 retinas) sorted in ascending order. There are significantly more somal pNF+ cells in Cx3cr1^gfp/gfp^ retinas (^*^p < 0.05; Wilcoxon ranked sum test). Solid horizontal line indicates the population mean, dashed lines indicate 1 standard deviation above and below the mean. Error bars represent the SEM. **(E)** There is significantly less expression of axonal transport genes Kif1b and Atp8a2 in Cx3cr1^gfp/gfp^ DBA/2J whole retina cDNA (25% and 15% less expression respectively, ^*^p < 0.05; Student's *t*-test; error bars represent the SEM, *n* = 8 DBA/2J retinas and 9 Cx3cr1^gfp/gfp^ DBA/2J retinas). Scale bars: 500 μm **(A)**; 50 μm **(B,C)**.

Since the FKN ligand is enriched in the ganglion cell layer of the retina in both humans and mice (Silverman et al., 2003; Zieger et al., 2014) including RGCs in the DBA/2J (Supplemental Figure 1), we reasoned that loss of FKN signaling may influence key aspects of RGC decline in glaucoma. Deficits in axonal transport progressively affect RGC axon and somal integrity in glaucoma (Soto et al., 2008; Crish et al., 2010; Dengler-Crish et al., 2014). One marker of disrupted axonal transport is the build-up of phosphorylated neurofilament (pNF; medium and heavy) proteins within the proximal axon segment, cell body, and dendrites of RGCs (Soto et al., 2008). Within the central retina of DBA/2J and Cx3cr1^gfp/gfp^ DBA/2J mice at 10–11 months of age we found regions with both low and high numbers of somal pNF+ RGCs (Figures 1A–C), consistent with the variable levels of pathology in the DBA/2J model. We observed an overall increase in the density of somal pNF+ RGCs in Cx3cr1^gfp/gfp^ DBA/2J vs. DBA/2J retinas (Figure 1D), with a significant shift in the distribution toward higher mean densities of somal pNF+ RGCs for Cx3cr1^gfp/gfp^ DBA/2J mice than in standard DBA/2J retinas (*p* < 0.05 by Kolmogorov-Smirnov test). This resulted in a significantly increased population mean density of somal pNF+ RGCs in Cx3cr1^gfp/gfp^ DBA/2J compared to the DBA/2J retinas (10.51 vs. 6.46 cells/mm^2^ respectively, Figure 1D; *p* < 0.05 by Wilcoxon rank sum test).

Since somal accumulation of pNF suggests deficits in axonal transport, we isolated whole retina mRNA and performed quantitative RT-PCR to determine whether there is altered expression of genes involved in axon transport. Previous work has shown reduced expression of the anterograde transport motor Kif1b within sectors of DBA/2J retinas depleted of Fluorogold positive RGCs (Panagis et al., 2010). Compared to DBA/2J, we found that expression of Kif1b was significantly reduced in Cx3cr1^gfp/gfp^ DBA/2J retinas (25% reduction; *n* = 9 retinas Cx3cr1^gfp/gfp^ DBA/2J, *n* = 8 DBA/2J; *p* < 0.05, Student's *t*-test, Figure 1E). Loss of the phosphatidyl-serine flippase, Atp8a2, also disrupts axonal transport with an increase in RGCs with somal pNF (Zhu et al., 2012). Expression of Atp8a2 by qRT-PCR was also significantly reduced in Cx3cr1^gfp/gfp^ DBA/2J vs. DBA/2J retinas (15% reduction; *n* = 9 retinas Cx3cr1^gfp/gfp^ DBA/2J, *n* = 8 DBA/2J; *p* < 0.05 Student's *t*-test, Figure 1E). Thus, we find multiple lines of evidence for increased axon transport dysfunction in the DBA/2J retina with loss of fractalkine signaling.

### Loss of Cx3cr1 in DBA/2J mice increases the number of early declining RGCs

**Figure 2:**
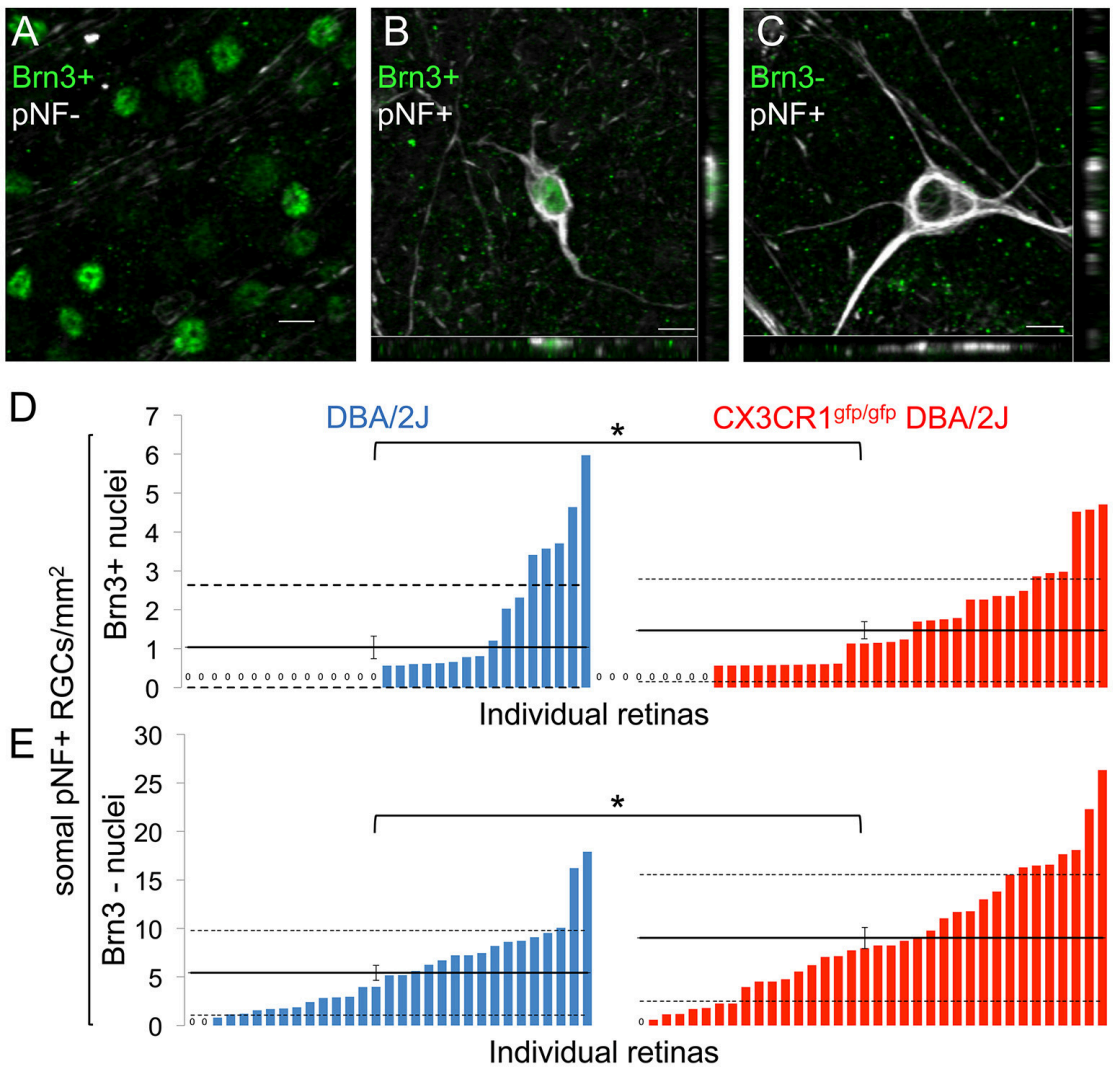
**Loss of fractalkine signaling results in increased numbers of RGCs in both early and late stages of decline. (A)** Confocal image of retinal flat mount showing Brn3+ nuclei that lack accumulation of somal pNF. **(B,C)** Confocal image of cells with somal accumulation of pNF, and either expression **(B)** or absence **(C)** of Brn3. Orthogonal views in the x-y (bottom) and y-z (side) planes are shown. **(D,E)** Distribution of the density of cells that are Brn3+/somal pNF+ **(D)** or Brn3-/somal pNF+ **(E)** within the central retina in DBA/2J (blue *n* = 31 retinas) and Cx3cr1^gfp/gfp^ DBA/2J (red *n* = 36 retinas) mice, sorted in ascending order. Cx3cr1^gfp/gfp^ DBA/2J retinas contain significantly more Brn3+/somal pNF+ cells (**D**, ^*^ *p* < 0.05; Wilcoxon ranked sum test) and Brn3-/somal pNF+ cells (**E**, ^*^p < 0.05; Wilcoxon ranked sum test) than DBA/2J retinas. Solid horizontal line indicates the population mean, dashed lines indicate 1 standard deviation above and below the mean. Error bars represent the SEM. Scale bars: 10 μm.

Since loss of fractalkine signaling resulted in increased numbers of somal pNF+ RGCs, we reasoned that this could be due to increased numbers of affected RGCs, impaired clearance of degenerating RGCs, or both. To determine whether the increase in pNF RGCs was due to impaired clearance we identified RGCs in earlier and later stages of degeneration. Early declining RGCs downregulate the expression of characteristic genes such as members of the Brn3 transcription factor family in response to damage (Schlamp et al., 2001; Huang et al., 2006; Buckingham et al., 2008; Soto et al., 2008). In the DBA/2J retina Brn3 downregulation precedes the clearance of the RGC soma by several weeks (Soto et al., 2008). We quantified subsets of RGCs with somal buildup of pNF with or without nuclear Brn3 expression in DBA/2J and Cx3cr1^gfp/gfp^ DBA/2J retinas. In both genotypes we identified three populations of RGCs at 10–11 months: healthy RGCs that express Brn3 and lack somal pNF (Brn3+/pNF-; Figure 2A); early declining RGCs that express Brn3 but accumulate somal/somatodendritic pNF (Brn3+/pNF+; Figure 2B); and RGCs in later stages of degeneration that lose Brn3 expression and retain somal/somatodendritic pNF (Brn3-/pNF+; Figure 2C) before ultimately being eliminated.

Quantification of the relative cell density for these three subsets of RGCs revealed a higher proportion of Cx3cr1^gfp/gfp^ DBA/2J retinas that had Brn3+/pNF+ RGCs compared to age-matched DBA/2J mice and a significant increase in the mean density of Brn3+/pNF+ RGCs (1.47 vs. 1.03 cells/mm^2^ respectively, Figure 2D; *p* < 0.05 by Wilcoxon rank sum test), demonstrating an increase in early declining RGCs in the absence of fractalkine signaling. We also measured a significant increase in the mean number of Brn3-/pNF+ RGCs in late stages of degeneration, consistent with the overall increase in pNF+ RGCs (9.02 vs. 5.43 cells/mm^2^ respectively, Figure 2E; *p* < 0.05 by Wilcoxon rank sum test). Since there was not a selective increase in Brn3-/pNF+ RGCs, we conclude that the increased density of RGCs with somal pNF buildup is not simply a failure to clear RGCs in late stages of decline in Cx3cr1^gfp/gfp^ DBA/2J retinas.

To further assess possible changes in clearance and cell death, we performed immunostaining for cleaved caspase 3, and found that none of the pNF+ cells were positive (0 out of 40 somal pNF+ RGCs, *n* = 2 retinas; Supplemental Figure 2). In addition, we observed that the nuclei of RGCs with somal pNF accumulation were not pyknotic (Supplemental Figure 3) suggesting they are not yet undergoing apoptosis. Since fractalkine has been shown to induce expression of the opsonin milk fat globule-EGF factor 8 (Mfge8), which stimulates myeloid cells to clear apoptotic neurons (Fuller and Van Eldik, 2008) we assessed levels of Mfge8 mRNA. However, we found no significant difference in Cx3cr1^gfp/gfp^ DBA/2J (*n* = 3) vs. DBA/2J (*n* = 3) retinas (*p* > 0.05, Student's *t*-test; data not shown). Together, these findings suggest that loss of fractalkine signaling is amplifying the generation of dystrophic RGCs rather than impairing their clearance.

### Loss of Cx3cr1 in the DBA/2J mouse does not alter Brn3-positive RGC numbers

**Figure 3:**
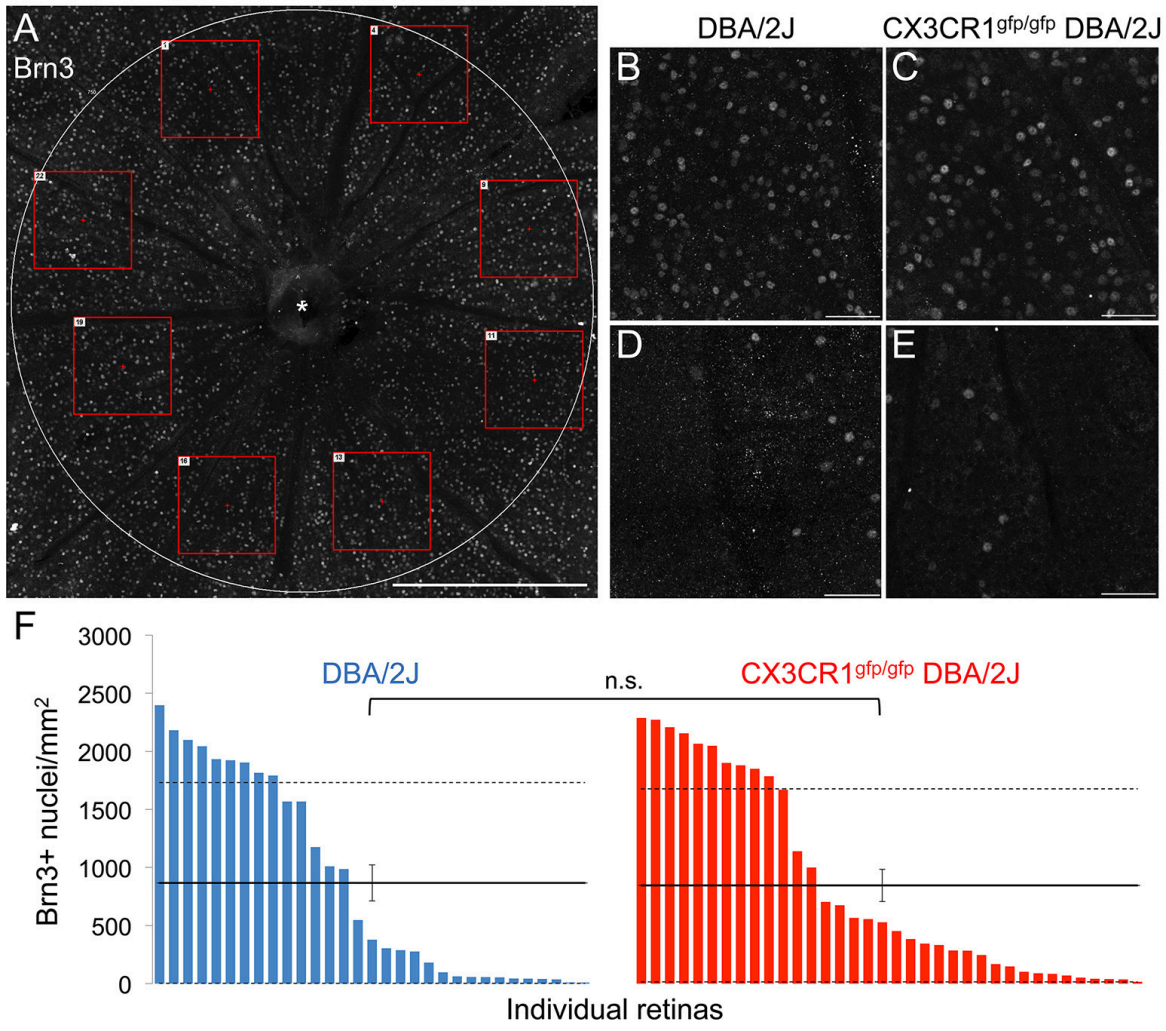
**The density of Brn3+ nuclei does not change with loss of fractalkine signaling. (A)** Confocal image of a representative Cx3cr1^gfp/gfp^ DBA/2J retinal flat mount showing the Brn3 immunofluorescence in the central retinal area (1.77 mm^2^). Red boxes depict examples of 0.06 mm^2^ fields sampled across 8 sectors. **(B–E)** Higher magnification images of 0.06 mm^2^ fields (250 × 250 μm) in retinas from DBA/2J **(B,D)** or Cx3cr1^gfp/gfp^ DBA/2J **(C,E)**, representative of high **(B,C)**, and low **(D,E)** Brn3+ nucleus density. **(F)** Distribution of Brn3+ nuclei densities per retina in DBA/2J (blue; *n* = 31 retinas) and Cx3cr1^gfp/gfp^ DBA/2J (red; *n* = 36 retinas), sorted from high to low density. Solid horizontal lines indicate population means, dashed lines indicate 1 standard deviation above and below the mean. Error bars represent the SEM (n.s.: *p* > 0.05; Wilcoxon ranked sum test). Asterisk indicates optic nerve head. Scale bars: 500 μm **(A)**; 50 μm **(B–E)**.

To determine if loss of Cx3cr1 affected other retinal readouts of RGC compartmentalized degeneration we assessed whether loss of fractalkine signaling altered the density of cells expressing Brn3 within the ganglion cell layer (Figure 3A). We quantified the density of Brn3+ nuclei in eight sectors for each retina (Figure 3A), and found that regardless of genotype, there were regions with high and low numbers of Brn3+ nuclei (Figures 3B–E), consistent with sectorial downregulation of Brn3 expression in both DBA/2J and Cx3cr1^gfp/gfp^ DBA/2J retinas. We then calculated average densities of Brn3+ nuclei for each retina (Figure 3F). There were similar maximum densities of Brn3+ nuclei in each genotype (2400/mm^2^ in DBA/2J and 2300/mm^2^ in Cx3cr1^gfp/gfp^ DBA/2J Figure 3F), and similar distribution of retinas with varying levels of Brn3 depletion (Figure 3F), consistent with the range of disease severity typically observed in the DBA/2J model (Buckingham et al., 2008). Likewise, the population mean densities of Brn3+ nuclei for each genotype were not significantly different (867 and 846 cells per mm^2^ respectively, Figure 3F; *p* > 0.05 Wilcoxon rank sum test). To rule out sampling artifacts, we counted all Brn3+ nuclei within the central retina (1.77 mm^2^) for a subset of retinas (*n* = 6 DBA/2J and 7 Cx3cr1^gfp/gfp^ DBA/2J with similar ranges of pathology), and obtained similar results (data not shown). Therefore, we conclude that at this timepoint, loss of fractalkine signaling did not affect the characteristic downregulation of Brn3 expression in RGCs that occurs in the DBA/2J retina with age.

### Loss of Cx3cr1 in DBA/2J mice does not exacerbate optic nerve degeneration

**Figure 4:**
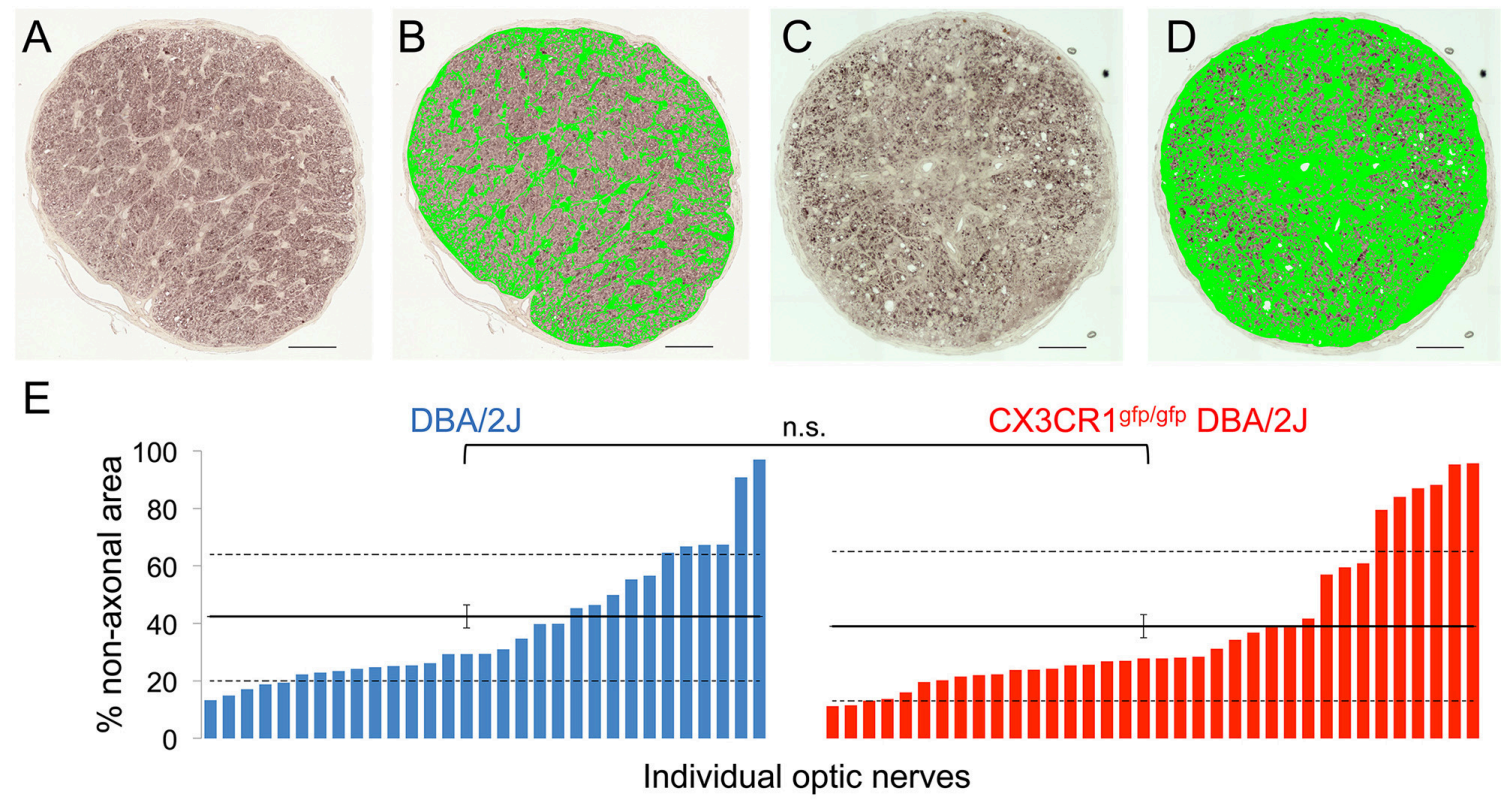
**There is no difference in the amount of gliosis and scarring in the optic nerve with loss of fractalkine signaling. (A)** Low magnification micrograph of a healthy optic nerve cross section with low gliosis and few dystrophic axonal profiles used for subsequent image analysis. **(B)** The same nerve in **(A)** after threshold-based segmentation of non-axonal area, including glia, and extracellular matrix but excluding blood vessels (binary mask in green). **(C)** Low magnification micrograph of a diseased optic nerve cross section with high gliosis and many dystrophic axonal profiles used for subsequent image analysis. **(D)** The same nerve in **(C)** after segmentation of the non-axonal areas, including gliotic and scar areas (binary mask in green). **(E)** Distribution of the non-axonal area percentages per optic nerve in DBA/2J (blue, *n* = 31) and Cx3cr1^gfp/gfp^ null DBA/2J (red *n* = 36) sorted in ascending order. There is no significant difference between the genotypes (n.s. *p* > 0.05; Wilcoxon ranked sum test). Horizontal lines indicate the population mean, dashed lines indicate 1 standard deviation above and below the mean. Error bars represent the SEM. Scale bars: 50 μm.

RGC degeneration in the DBA/2J model is well characterized within the optic nerve to include a progressive loss of RGC axons and a subsequent replacement by glia and extracellular matrix (John et al., 1998; Libby et al., 2005a; Schlamp et al., 2006; Lye-Barthel et al., 2013; Bosco et al., 2015a,b, 2016; Cooper et al., 2016). To determine if the potential impact on axonal transport by loss of Cx3cr1 affected optic nerve degeneration, we estimated the relative coverage of non-axonal area using segmentation of glia and extracellular matrix in nerve cross-sections (Bosco et al., 2015a,b, 2016). For both the DBA/2J and Cx3cr1^gfp/gfp^ DBA/2J genotypes at 10–11 months, we observed a similar distribution of nerves with non-axonal area ranging from 10 to above 95% in individual nerves (Figures 4A–E). We did not observe a statistically significant difference in the mean non-axonal area for Cx3cr1^gfp/gfp^ DBA/2J optic nerves as compared to DBA/2J (42 and 39% respectively; Figure 4E; *p* > 0.05 by Wilcoxon rank sum test). To confirm these findings, we used a qualitative scoring method (Libby et al., 2005a) on a subset of 24 nerves of each genotype, and did not find a significant difference in optic nerve degeneration between the genotypes (Chi-squared value of 0.93; data not shown). Therefore, we conclude that loss of fractalkine signaling did not alter proximal optic nerve degeneration on the DBA/2J background at this age.

### Macrophage infiltration is increased in Cx3cr1^gfp/Gfp^ DBA/2J retinas

**Figure 5:**
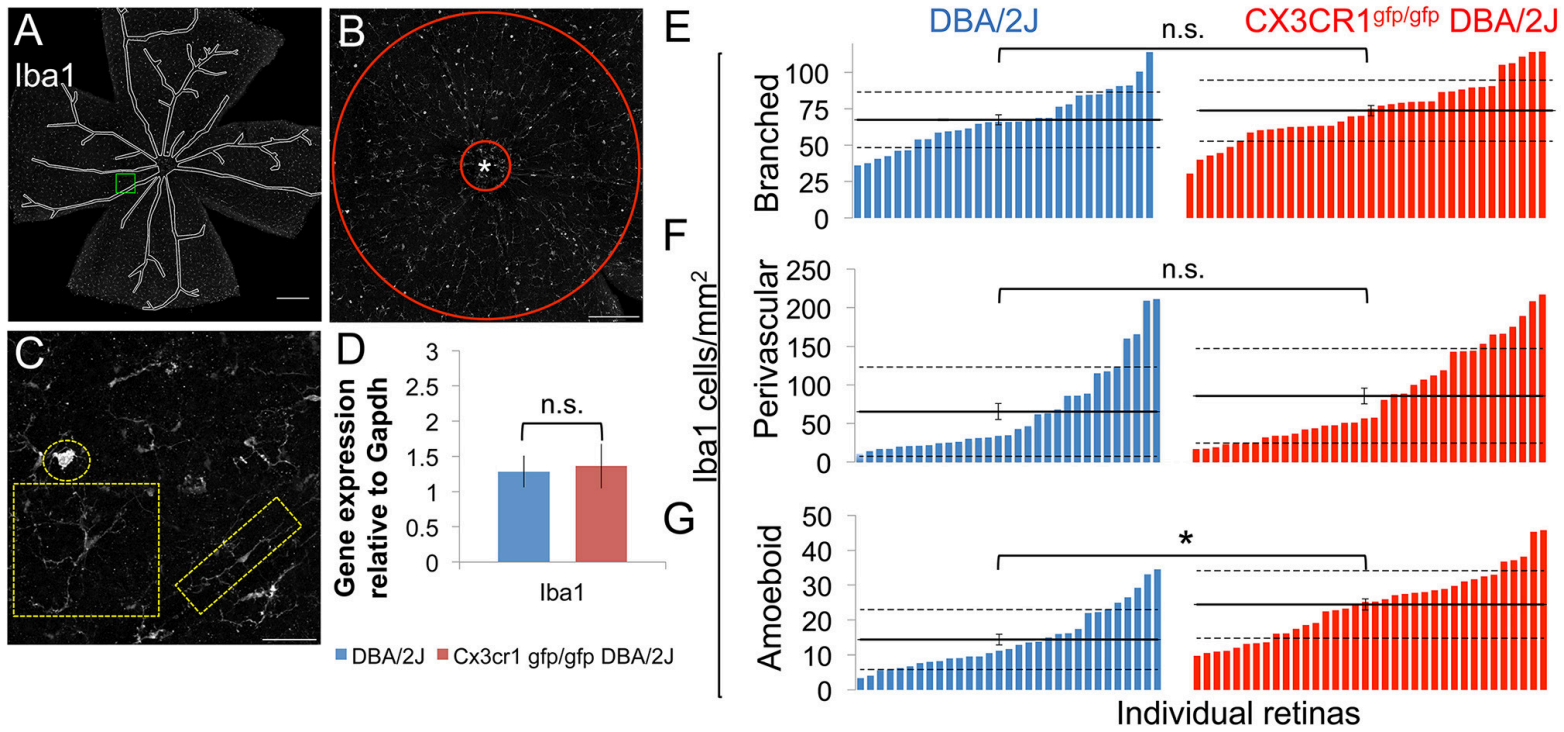
**Loss of fractalkine signaling results in greater numbers of amoeboid shaped Iba1+ cells. (A)** Low magnification view of an entire retinal flat mount that was sampled for Iba1+ cells. Blood vessels (traced) were excluded from analysis. **(B)** Low magnification image of the central retina (1.77 mm^2^) depicting the area sampled for branched and perivascular shaped Iba1+ cells excluding the optic nerve head (marked by an asterisk and traced by the red circle). **(C)** Higher magnification view of the area indicated in A demonstrating the 3 categories of shapes for Iba1+ cells, showing an amoeboid morphology (circle), complex branching (square), or perivascular distribution (rectangle). **(D)** There is no difference in the expression of Iba1 in Cx3CR1^gfp/gfp^ DBA/2J retinas (n.s., ^*^: *p* > 0.05; Student's *t*-test; error bars represent the SEM, 5 retinas per condition). **(E–G)**: Distribution of the number of branched **(E)**, perivascular **(F)**, and amoeboid shaped Iba1+ cells **(G)** within the central retina in DBA/2J (blue, *n* = 31) and Cx3cr1^gfp/gfp^ DBA/2J (red *n* = 36) sorted in ascending order. There are significantly more amoeboid Iba1+ cells (1.7-fold ^*^p < 0.05; Wilcoxon ranked sum test) in Cx3cr1^gfp/gfp^ retinas. There are no statistically significant changes in the numbers of branched or perivascular Iba1+ cells (n.s. *p* > 0.05; Student's *t*-test and Wilcoxon ranked sum test respectively). Horizontal lines indicate the population mean, dashed lines indicate 1 standard deviation above and below the mean. Error bars represent the SEM. Scale bars: 500 μm **(A)**, 250 μm **(B)**, 50 μm **(C)**.

Given that fractalkine signaling regulates many myeloid cell responses, including microglial activation, as well as peripheral monocyte recruitment and distribution (Cardona et al., 2006; Sennlaub et al., 2013; Wolf et al., 2013; Limatola and Ransohoff, 2014), we examined how loss of the fractalkine receptor affected myeloid cells in terms of morphology, number, distribution, and levels of the activation-associated gene, Iba1 in 10–11 month-old Cx3cr1^gfp/gfp^ DBA/2J vs. DBA/2J retinas. We observed three distinct morphological subtypes of Iba1+ cells within the retina: cells with a small soma and many processes (“branched”), cells with two main processes on polar opposite sides typically outlining blood vessels (“perivascular”), and spherical cells with no processes (“amoeboid”) (Figures 5A–C). We presume these Iba1+ cell subtypes to respectively represent resident parenchymal microglia, perivascular macrophages, and either infiltrating macrophages or highly activated resident microglia. We found no change in the density of branched Iba1+ cells (Figure 5E; *p* > 0.05 by Student's *t*-test) between Cx3cr1^gfp/gfp^ DBA/2J retinas compared to DBA/2J retinas, suggesting no change in the resident microglia population. There was a trend toward an increase in the mean density of perivascular cells in Cx3cr1^gfp/gfp^ DBA/2J retinas (Figure 5F, *p* < 0.1 by Wilcoxon rank sum test), and a significant 1.7-fold increase in the mean density of amoeboid Iba1+ cells (Figure 5G, *p* < 0.05 by Wilcoxon rank sum test).

**Figure 6:**
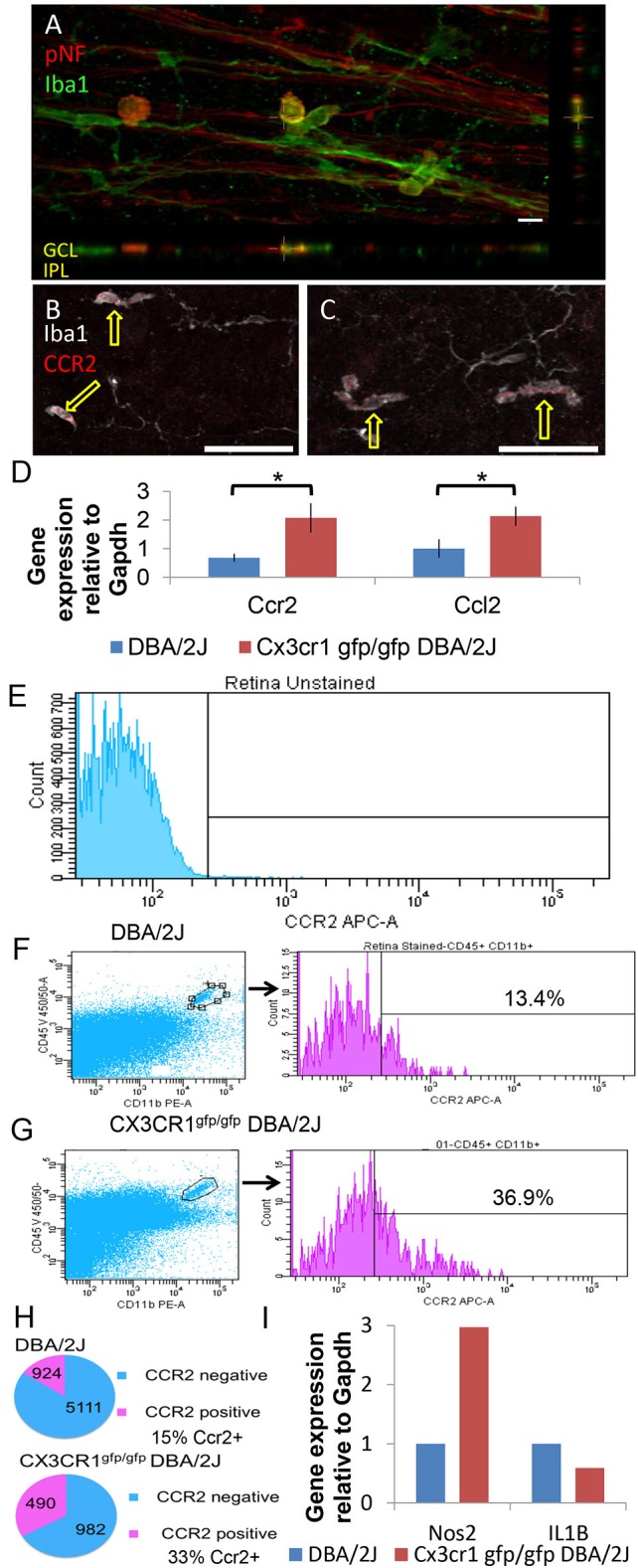
**Amoeboid Iba1+ cells are peripherally derived macrophages. (A)** Amoeboid Iba1+ cells localize to the neurofibrillary layer. Volume view of a DBA/2J retinal whole mount stained for the axonal marker pNF (red) and the microglia/macrophage marker Iba1 (green). Orthogonal views shown below and to the right. Amoeboid Iba1+ cells showed non-specific reactivity with the donkey anti-mouse secondary antibody used to detect pNF (not shown). **(B)** Single z-plane (0.8 μm) from a control DBA/2J retinal whole mount immunostained for the peripheral macrophage marker Ccr2 (red) and Iba1 (white). The amoeboid shaped Iba1+ cells are positive for Ccr2 (arrows) while branched Iba1+ cells are negative. **(C)** Single z-plane (0.8 μm) from a Cx3cr1^gfp/gfp^ DBA/2J retina immunostained for the peripheral macrophage marker Ccr2 (red) and Iba1 (white). As in **(B)** the amoeboid shaped Iba1+ cells are positive for Ccr2 (arrows) while branched Iba1+ cells are negative. **(D)** There is significantly more expression of Ccr2 and Ccl2 in Cx3cr1^gfp/gfp^ DBA/2J whole retina cDNA (3 and 2-fold more expression respectively, ^*^p < 0.05; Student's *t*-test; error bars represent the SEM, 5 retinas per condition). **(E)** Gating parameters were set by dissociated retinal cells not exposed to antibodies. 56,935 viable cells collected. **(F)** Left, representative plot of DBA/2J assessing CD45^+^ CD11b^+^ (gate shown) from 129,000 to 200,000 viable cells. Right, histograms and percent CCR2^+^ of gated CD45^+^CD11b^+^ population. **(G)** Left, representative plots of Cx3cr1^gfp/gfp^ DBA/2J assessing CD45^+^ CD11b^+^ (gate shown) from 129,000 to 200,000 viable cells as in F. Right, histograms and percent CCR2^+^ of gated CD45^+^CD11b^+^ population. **(H)** Top, total number and percent of CCR2^+^ and CCR2^−^ of all CD45^+^CD11b^+^ cells analyzed by flow cytometry in DBA/2J *n* = 8 pooled retinas (293,780 cells collected). Bottom, total number and percent of CCR2^+^ and CCR2^−^ of all CD45^+^CD11b^+^ cells analyzed by flow cytometry in Cx3cr1^gfp/gfp^ DBA/2J *n* = 6 pooled retinas (453,727 cells collected). **(I)**: There is ~3-fold more expression of Nos2 but 33% less expression of IL1β in FACS sorted myeloid cells (*n* = 8 DBA/2J pooled retinas and *n* = 6 CX3CR1^gfp/gfp^ DBA/2J pooled retinas). Scale bars: 50 μm **(B,C)** 10 μm **(A)**.

To determine whether the amoeboid Iba1+ cells were infiltrating macrophages, we first confirmed their exclusive localization to the nerve fiber layer by volumetric view of confocal image stacks (Figure 6A), a distribution previously reported for infiltrating macrophages in retinal injury paradigms (Garcia-Valenzuela and Sharma, 1999) and for macrophages that patrol the vitreous body (Vagaja et al., 2012). Furthermore, to positively identify these amoeboid Iba1+ cells as peripheral monocytes and not as microglia, we immunostained retinas for the peripheral macrophage marker CCR2 (Saederup et al., 2010). We determined that the amoeboid shaped Iba1+ cells stained positive for CCR2 in five whole mount DBA/2J retinas (Figure 6B) and in two whole mount Cx3cr1^gfp/gfp^ DBA/2J retinas (Figure 6C), confirming their identity as peripherally derived macrophages. Consistent with this, qRT-PCR analysis showed a 3-fold increase in levels of Ccr2 expression in Cx3cr1^gfp/gfp^ DBA/2J retinas (*n* = 5) vs. DBA/2J retinas (*n* = 5; *p* < 0.05; Student's *t*-test, Figure 6D). There was a concurrent 2-fold increase in levels of the ligand Ccl2 which can promote macrophage infiltration (*n* = 5 each; *p* < 0.05; Student's *t*-test, Figure 6D).

To selectively sort for myeloid cell types present in the DBA/2J retina, and further confirm an increase in peripherally-derived macrophages in the absence of fractalkine signaling, we performed flow cytometry analysis on Cx3cr1^gfp/gfp^ DBA/2J (*n* = 6) and DBA/2J (*n* = 8) retinas, evaluating CD11b^+^, CD45^+^, CCR2 ± cells (Figures 6E–G). CCR2+ cells account for approximately 15% of the CD11b^+^ CD45^+^ double positive myeloid population in 12 month-old DBA/2J retinas, while they represent about 33% in age-matched Cx3cr1^gfp/gfp^ DBA/2J retinas (Figure 6H). Thus, the CCR2+ cells, which represent peripherally-derived macrophages, are more than doubled in Cx3cr1^gfp/gfp^ DBA/2J retinas, which is consistent with the observed increase in amoeboid Iba1+ cells in the retinas lacking Cx3cr1 (Figure 5G).

### Loss of FKN signaling results in selective changes in myeloid cell gene expression

To determine how loss of fractalkine signaling in myeloid cells might impact pathways involved in RGC degeneration, we assessed changes in expression of genes associated with myeloid cell activation between Cx3cr1^gfp/gfp^ DBA/2J and DBA/2J retinas. qRT-PCR analysis of whole retina RNA detected equal levels of Iba1 mRNA between the genotypes (*n* = 5 each, Figure 5D), suggesting no significant changes in the overall levels of myeloid cell activation. We also assessed changes in expression of the proinflammatory gene Il1β in Cx3cr1^gfp/gfp^ DBA/2J and DBA/2J retinas by qRT-PCR and found a 2.34-fold increase, but this was not significant (*n* = 6 each genotype; *p* = 0.1 by Student's *t*-test, data not shown). Since loss of Cx3cr1 has been shown to increase expression of Il1β [26, 57 we performed qRT-PCR on FACS-sorted CD11b+ CD45+ cells (Figures 6E–G). In contrast to whole retina mRNA levels, Il1β was reduced by 33% in myeloid cells from Cx3cr1^gfp/gfp^ DBA/2J (*n* = 6) retinas vs. DBA/2J (*n* = 8) retinas, suggesting that this population is not contributing to increased expression of this gene in the DBA/2J retina.

Since reactive nitric oxide (NO) from activated microglia can induce axon transport deficits (Stagi et al., 2005), we performed qRT-PCR analysis to assess changes in the expression of Nos-2, which contributes to the production of NO. We found a 1.89-fold increase in Nos-2 expression in Cx3cr1^gfp/gfp^ DBA/2J and DBA/2J whole retinas, which was not significant (*n* = 6 each genotype; *p* = 0.1 by Student's *t*-test, data not shown), but when we enriched for myeloid cells by FACS-sorting CD11b+ CD45+ cells (Figures 6D–F) we observed a 3-fold increase in Nos-2 in myeloid cells from Cx3cr1^gfp/gfp^ DBA/2J (*n* = 6) retinas vs. DBA/2J (*n* = 8) retinas (Figure 6I). Thus, we find selective changes in the expression of genes in myeloid cells consistent with a role in disrupting axon transport.

## Discussion

### Loss of Cx3cr1 affects compartmentalized RGC degeneration

We found that loss of Cx3cr1 in the DBA/2J model of chronic glaucoma had a selective effect on somal accumulation of pNF in RGCs in 10–11 month-old mice, suggesting deficits in axonal transport. These effects were uncoupled from other RGC degenerative changes, including loss of Brn3 gene transcription or optic nerve histopathology. We also observed increased infiltration of CCR2+ peripheral macrophages, but no change in myeloid cell activation overall, since resident microglia were not altered in density, or morphology, and overall levels of Iba1 mRNA were unaffected. Our findings suggest that distinct mechanisms may contribute to different aspects of RGC decline in glaucoma, with axonal transport selectively altered after loss of Cx3cr1 in myeloid cells such as microglia and macrophages.

### Loss of Cx3cr1 enhances axon transport deficits in RGC neurodegeneration

We observed that loss of Cx3cr1 resulted in enhanced somal accumulation of pNF, as well as reduced expression of the anterograde transport motor Kif1b, consistent with exacerbation of axonal transport deficits. There is a correlate in humans where mutations in KIF1A affect axonal transport and can lead to progressive neurodegeneration, including optic atrophy (Okamoto et al., 2014). Decline in axonal transport is an early and prevalent feature of many forms of neurodegeneration, including glaucoma, generally preceding signs of structural axon degeneration (Pease et al., 2000; Quigley et al., 2000; De Vos et al., 2008; Soto et al., 2008; Crish et al., 2010, 2013; Chidlow et al., 2011; Millecamps and Julien, 2013; Dengler-Crish et al., 2014). Our findings are consistent with previous studies showing that neuroinflammation, including microglia activation and oxidative stress, is a contributing factor to early axon transport dysfunction (Takeuchi et al., 2005). In an animal model of multiple sclerosis, which is characterized by significant neuroinflammation, axon transport deficits were found to be an early state of axonal dysfunction. These deficits preceded structural changes to axons and could be reversed by anti-inflammatory as well as anti-oxidant treatment (Sorbara et al., 2014).

We observed increased expression of Nos-2 in retinal DBA/2J myeloid cells lacking Cx3cr1. Reactive nitric oxide (NO) from microglia can focally induce axon transport deficits *in vitro* (Stagi et al., 2005), and promote axonal damage *in vivo* (Nikic et al., 2011). Nos-2 is not required for RGC death and axon degeneration in DBA/2J mice (Libby et al., 2007), but effects on axon transport have not been assessed. Therefore, it is possible that there is a selective role for this pathway in this aspect of neuronal decline, consistent with our findings. Notably, alleviating oxidative stress in the DBA/2J model with alpha-lipoic acid treatment results in improved axonal transport (Inman et al., 2013).

### Optic nerve degeneration and Brn3 downregulation are not affected by loss of Cx3cr1

While we observed an increase in somal accumulation of pNF, there was no change in structural degeneration of the optic nerve in Cx3cr1^gfp/gfp^ DBA/2J mice relative to DBA/2J mice. This may be due to the fact that structural decline of axons can lag changes in transport, as shown in a model of multiple sclerosis (Sorbara et al., 2014). Consistent with this, there is structural persistence in the optic pathway well after axon transport failure in an ocular hypertension model of glaucoma (Crish et al., 2010; Crish and Calkins, 2011). Notably, in glaucoma patients, functional decline of RGCs as measured by pattern electroretinography (PERG) significantly precedes structural changes, such as loss of nerve fiber layer thickness (Banitt et al., 2013), and in animal models reduced PERG amplitude can result from blockade of axon transport (Chou et al., 2013). Alternatively, structural degeneration of axons may be independently regulated, for example by calpain-dependent cleavage of cytoskeletal components (Crish and Calkins, 2011; Wang et al., 2012).

Interestingly, there are other instances of somal accumulation of pNF in RGCs being uncoupled from optic nerve degeneration. The wabbler-lethal mutant mouse has a loss of function mutation in the phosphatidyl-serine flippase, Atp8a2 that is involved in apoptosis and vesicle trafficking (Zhu et al., 2012; van der Mark et al., 2013). This mutant mouse shows disrupted axonal transport with an increase in RGCs with somal pNF, without observed increased damage to the optic nerve (Zhu et al., 2012). Since we found a significant reduction in expression of Atp8a2 in Cx3cr1^gfp/gfp^ DBA/2J vs. DBA/2J retinas, it is possible that loss of this pathway may be in part responsible for mediating the reduced RGC axonal transport phenotype. These results raise the question of whether exoplasmic-facing phosphatidyl-serine signaling to receptors on microglia or macrophages affects RGC degeneration, or if a cell autonomous role in membrane fluidity affects axonal transport.

In our study, Brn3 downregulation was also similar in both Cx3cr1^gfp/gfp^ DBA/2J and DBA/2J retinas. This suggests the density of RGCs was not significantly different with loss of Cx3cr1, and that Cx3cr1 signaling does not influence this early aspect of RGC degeneration. Thus, different aspects of RGC decline may occur at different rates or be regulated by distinct mechanisms. Consistent with this, deficiency in the dual leucine kinase (Dlk) signaling pathway alters somal but not axonal RGC degeneration (Fernandes et al., 2014). In contrast to our findings, significant reductions in beta tubulin III labeled RGCs were observed in a transient ocular hypertension model of glaucoma in Cx3cr1^gfp/gfp^ C57Bl6/J mice (Wang et al., 2014). Thus, it is possible that Cx3cr1 signaling has different effects in an acute injury vs. chronic model of RGC degeneration. Overall, our findings suggest that loss of fractalkine signaling selectively affects axon transport, providing evidence that distinct pathways mediate specific aspects of compartmentalized neurodegeneration.

### Loss of Cx3cr1 does not alter microglial activation

FKN signaling has been found to be a brake on microglial activation in many models of neurodegeneration (Cardona et al., 2006; Wolf et al., 2013; Limatola and Ransohoff, 2014). However, we found that the activation of the presumed resident microglia (branched, parenchymal Iba1+ cells) remained unchanged in terms of morphology and Iba1 mRNA levels with loss of Cx3cr1. A similar lack of change in microglia activation in Cx3cr1 knockouts was also observed in other disease models, such as prion-infected mice (Striebel et al., 2016). Activated microglia can secrete pro-inflammatory cytokines like Il1β (Patterson, 2015), and loss of Cx3cr1 has been shown to elevate levels of the pro-inflammatory cytokine Il1β (Cardona et al., 2006) In diabetic retinopathy, only a few genes associated with inflammation are significantly upregulated in Cx3cr1 knockout retina, including Il1β (Cardona et al., 2015). However, while we found that Nos2 was upregulated, as discussed above, we did not observe a significant increase in Il1β with loss of Cx3cr1 in the DBA/2J retina or in sorted myeloid cells including microglia. Thus, we conclude that overall levels of myeloid cell activation were not altered by loss of Cx3cr1 in this model, but that specific genes and pathways may be affected.

Other aspects of microglia function may also be affected by loss of Cx3cr1. For example, Cx3cr1 null microglia have been noted to more slowly remodel their processes (Liang et al., 2009), suggesting functional impairment. Whether this occurs in our model remains unclear. We also observed a perivascular population of Iba1+ cells that frequently lined up along axons (Figure 6A) in a manner reminiscent of that seen by CD11c+ dendritic cells in the optic nerve crush model of axonal injury (Lehmann et al., 2010; Heuss et al., 2014). Whether these cells represent a second niche of dendritic cells in the retina (Heuss et al., 2014) or a subpopulation of microglia (Dando et al., 2016) is unclear, but the proximity to RGC axons and retinal vessels suggest these cells would be a fruitful target in glaucoma research.

### Loss of Cx3cr1 promotes enhanced macrophage infiltration

While we did not observe obvious changes in microglia activation, loss of Cx3cr1 clearly resulted in enhanced Ccr2+ monocyte/macrophage infiltration. Consistent with this, we also found increased levels of expression of the ligand for Ccr2, Ccl2, which can promote infiltration (Sennlaub et al., 2013). Ccl2 was also found to be increased in brains from prion-infected Cx3cr1 knockout vs. wild type C57Bl6/J mice (Striebel et al., 2016). Increased CCR2+ macrophage infiltration on a Cx3cr1 null background is consistent with what has been observed in age and light damage models of subretinal inflammation that clearly showed a pathogenic role for these infiltrating macrophages (Sennlaub et al., 2013). However, since macrophages have been implicated in RGC regeneration (Cui et al., 2009; Yin et al., 2009), it is unclear whether their role in RGC compartmentalized degeneration is beneficial or detrimental. However, the increase in Ccr2+ macrophages in Cx3cr1^gfp/gfp^ DBA/2J retinas makes them a candidate for impacting axonal transport in RGCs since the numbers of resident microglia and perivascular macrophages were unchanged between the genotypes. Since differences do exist between the DBA/2J and acute models of IOP elevation, such as microbead injection (Sappington et al., 2010) or injection of hypertonic saline into episcleral veins (Morrison et al., 2011; Johnson et al., 2014) it may prove fruitful to evaluate the role of microglia and macrophages in these models.

Overall, our findings indicate that loss of Cx3cr1 increased the infiltration of CCR2+ macrophages into the DBA/2J retina, and selectively increased axonal transport dysfunction in this mouse model of chronic glaucoma, potentially driven by increased Nos2 expression in Cx3cr1-null myeloid cells. Thus, alterations in myeloid function may contribute to the impaired axonal transport seen early in many neurodegenerative diseases (Millecamps and Julien, 2013).

## Author contributions

MV, AB, and KB conceived the study and participated in its design and coordination. KB, MS, SA, and DC performed experiments and acquired data. KB and SA performed the analysis, and KB, SA, AB, and MV interpreted the data. KB and MV wrote the manuscript. All authors read and approved the final manuscript.

## Funding

This work was supported by National Institutes of Health grants 1R01EY023621 and 1R01EY020878 to MV, and grants from the Glaucoma Research Foundation, and Melza M. and Frank Theodore Barr Foundation to MV and DC.

### Conflict of interest statement

The authors declare that the research was conducted in the absence of any commercial or financial relationships that could be construed as a potential conflict of interest.

## Abbreviations

RGC: retinal ganglion cell
pNF: phosphorylated neurofilament
RT-PCR: reverse transcriptase polymerase chain reaction
Gapdh: glyceraldehyde 3-phosphate dehydrogenase
FACS: fluorescence-activated cell sorting
PBS: phosphate buffered saline
PPD: paraphenylenediamine
Iba1: ionized calcium binding adaptor molecule 1
Nos-2: nitric oxide synthase 2
NO: nitric oxide
Il1β: interleukin 1 beta
Mfge8: milk fat globule-EGF factor 8 protein
Dlk: dual leucine kinase
Ccl2: chemokine C-C motif ligand 2
Ccr2: chemokine C-C motif receptor 2
Cx3cl1: fractalkine
Cx3cr1: fractalkine receptor
GFP: green fluorescent protein
Cx3cr1^gfp/gfp^: Cx3cr1 -gfp knockin.
