# Supplementary material for: Loss of Fractalkine Signaling Exacerbates Axon Transport Dysfunction in a Chronic Model of Glaucoma
Source: Front Neurosci. 2016 Nov 24;10:526. doi: 10.3389/fnins.2016.00526 (PMC5123443; doi:10.3389/fnins.2016.00526)
Supplement: Supplementary file 1 [file Image1.pdf]

Supplementary Material

Loss of fractalkine signaling exacerbates axon transport dysfunction in a chronic model of glaucoma

Breen KT, Anderson SR, Steele MR, Calkins DJ, Bosco A, and Vetter ML*

*** Correspondence:** Vetter ML: monica@neuro.utah.edu

Supplemental Figure Legends:

Supplemental Figure 1. Fractalkine is highly expressed in the ganglion cell layer
(A): Radial cryosection through a 2 month Thy1$^{+/cfp}$ DBA/2J retina hybridized with a Cx3cl1 antisense probe depicting strong signal in RGCs and displaced amacrine cells in the ganglion cell layer (GCL). There is less expression in the inner (INL) nuclear layer in the retina. Left inset: Phase image of the black box indicated in A. Right inset: CFP channel (green) from the same field as the left inset showing a CFP-positive RGC.
(B): Subsequent section from the same animal as in A hybridized with a Cx3cl1 sense control probe, imaged, and developed identically.
Scale bar: 20 um.

Supplemental Figure 2. Somal pNF+ RGCs are not undergoing apoptosis.
(A-C): Radial cryosections through a Cx3CR1$^{gfp/gfp}$ DBA/2J retina double immunostained for pNF (white) and cleaved caspase 3 (red) with DAPI nuclear counterstain. Somal pNF+ RGCs lack activated caspase 3 (arrow).
Scale bar: 10 μm (A-C).

Supplemental Figure 3. Somal pNF+ RGCs do not have pyknotic nuclei.
(A-C): Confocal image of a Cx3CR1$^{gfp/gfp}$ DBA/2J retinal flat mount immunostained for pNF (B and C) counterstained for nuclear DAPI (A and C), shown as single (A and B) and merged channels (C). Nuclei appear normal throughout the GCL, even in RGCs with somal pNF accumulation (arrows).
Scale bar: 10 μm (A-C).

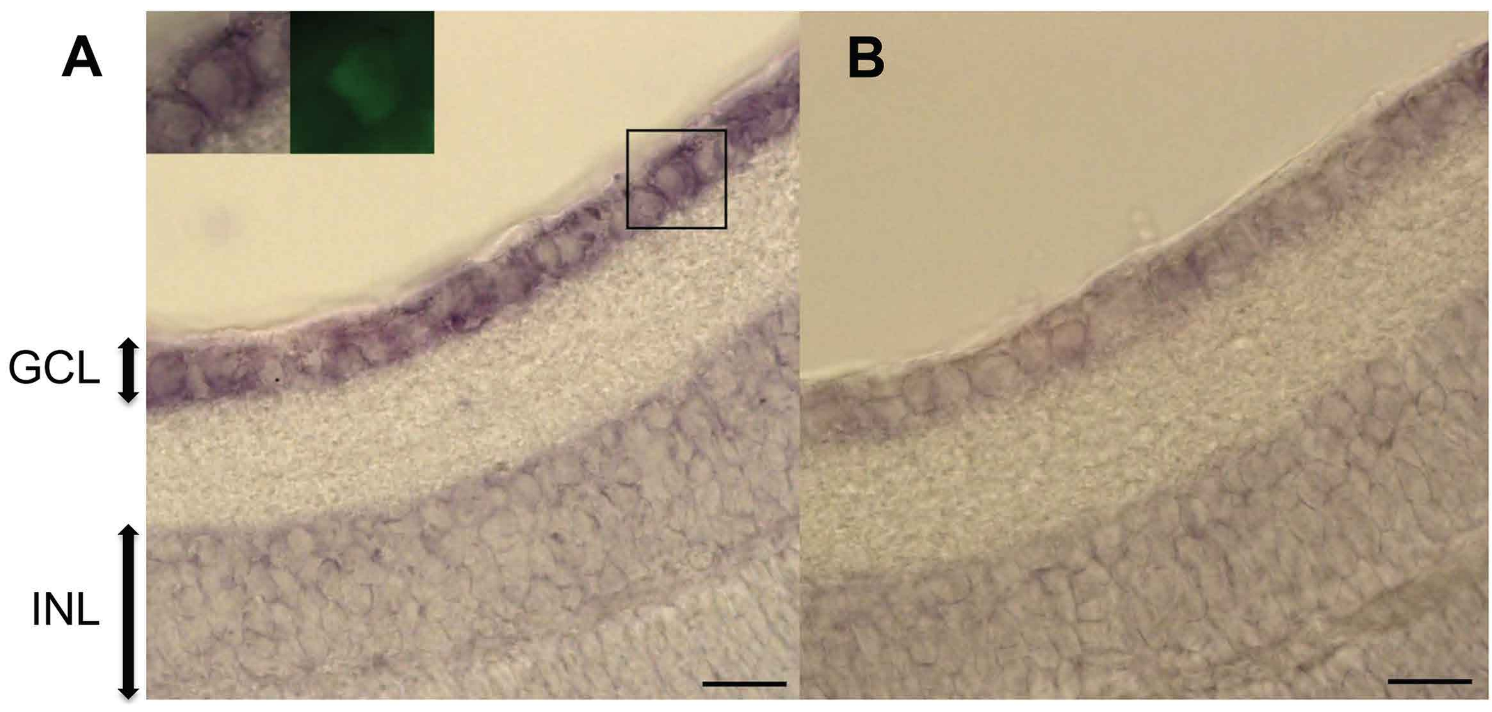

Suppl Fig 1

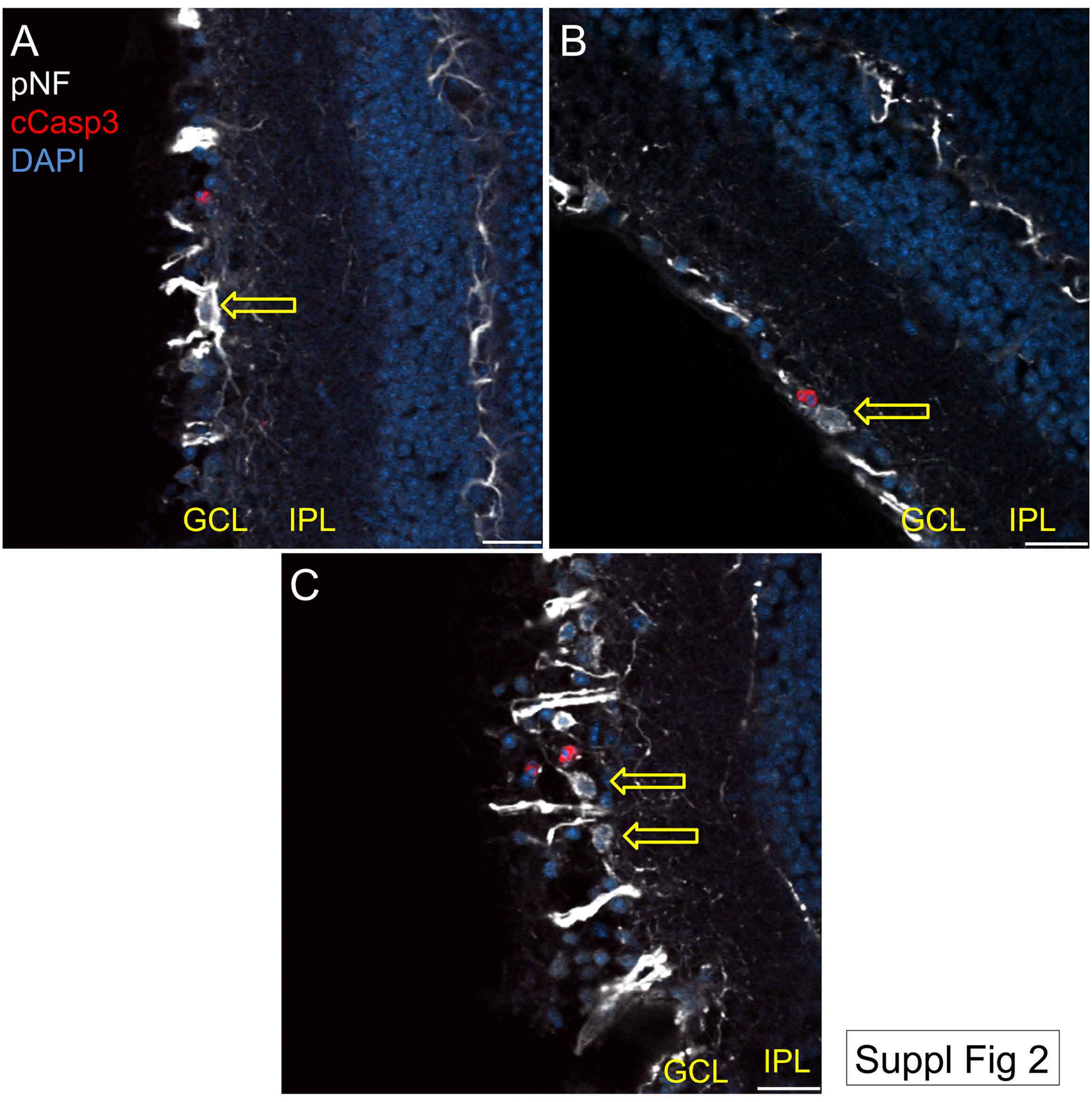

Suppl Fig 2

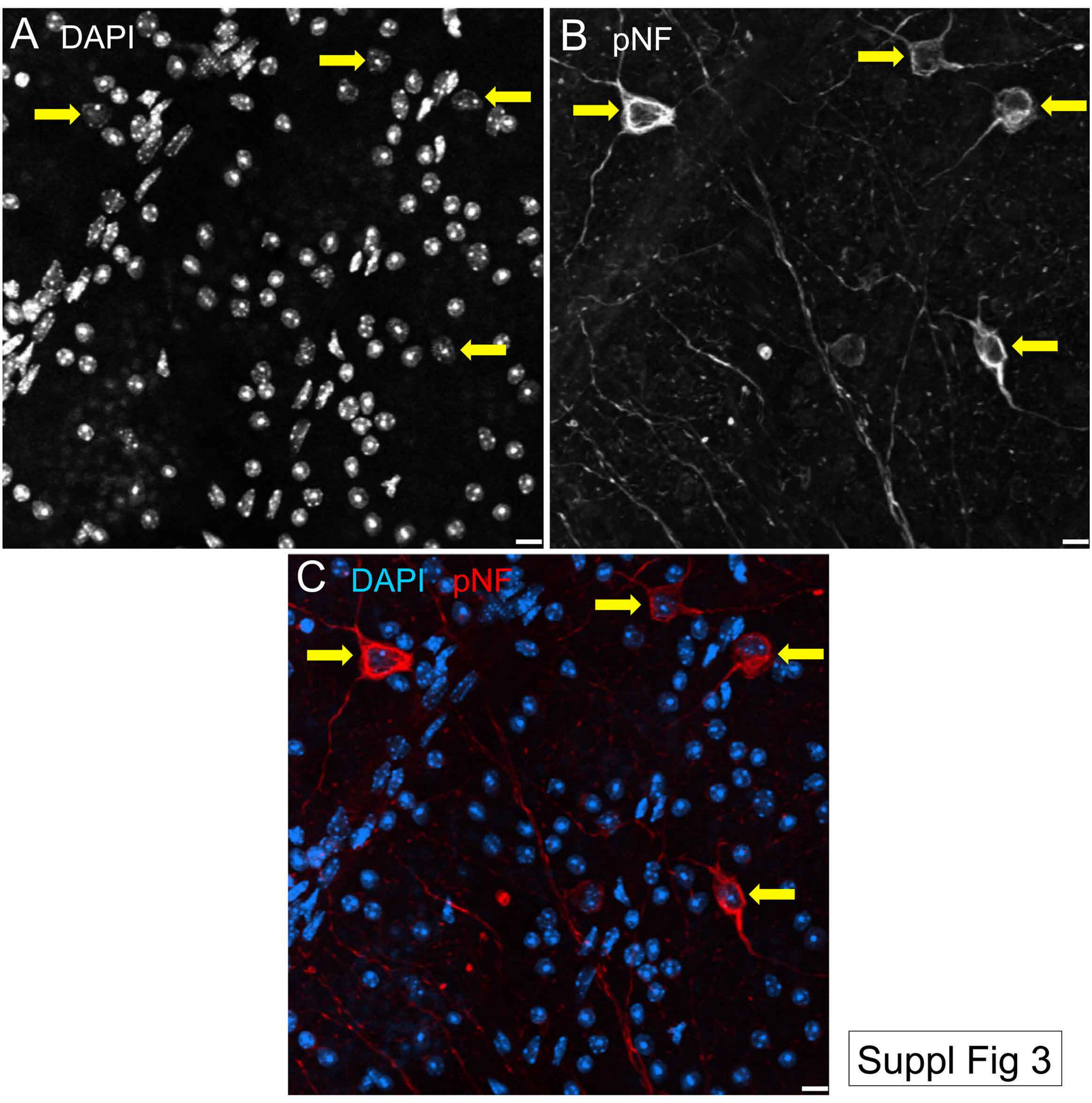

Suppl Fig 3
